# Ecological Factors Associated With Psychological Distress Among Adolescents: The Role of Educational Stress, Peer Victimization, and Physical Activity

**DOI:** 10.1002/brb3.71450

**Published:** 2026-04-29

**Authors:** Firoj Al‐Mamun, Tasmin Sayeed Nodi, Abu Hasnat Abdullah, Susmita Hossain, Md. Mehedee Hasan, Jyotie Malakar, Maysa Mohamed Rabea Abdelall, Mohammed A. Mamun

**Affiliations:** ^1^ CHINTA Research Bangladesh Dhaka Bangladesh; ^2^ Department of Public Health University of South Asia Dhaka Bangladesh; ^3^ Department of Public Health Daffodil International University Dhaka Bangladesh; ^4^ Bangladesh Dental College Dhaka Bangladesh; ^5^ Department of Maternal and Child Health National Institute of Preventive and Social Medicine Dhaka Bangladesh; ^6^ Department of Physical Sport Sciences College of Sport Sciences and Physical Activity Princess Nourah bint Abdulrahman University Riyadh

**Keywords:** adolescent mental health, ecological model, educational stress, peer victimization, physical activity, psychological distress

## Abstract

**Background:**

Adolescent psychological distress is shaped by influences across educational, familial, peer, and behavioral domains, yet few studies in low‐ and middle‐income countries (LMICs) have examined these determinants within a unified theoretical framework. Guided by Bronfenbrenner's bioecological model, this study investigated ecological factors associated with psychological distress among Bangladeshi adolescents, with particular attention to educational stress, peer victimization, and physical activity.

**Methods:**

A school‐based cross‐sectional survey was conducted among 501 higher secondary students in Bangladesh. Psychological distress was assessed using the Depression Anxiety and Stress Scale‐21, whereas educational stress, family relationship quality, peer relationships, and physical activity were measured using validated instruments. Hierarchical multiple linear regression models evaluated the incremental contribution of theoretically ordered factor blocks. Relative importance analysis (Lindeman–Merenda–Gold method) quantified each factor's proportional contribution to explained variance. Domain‐specific regression models were conducted for depression, anxiety, and stress. Heteroskedasticity‐consistent (HC3) robust standard errors were applied.

**Results:**

The fully adjusted model explained 23.8% of the variance in overall psychological distress. Educational stress (*B* = 0.56, *p* < 0.001), female gender (*B* = 12.32, *p* < 0.001), and peer victimization (*B* = 2.07, *p* < 0.001) were positively associated with distress, whereas physical activity was inversely associated (*B* = −0.91, *p* = 0.004). Relative importance analysis indicated that educational stress (20.4%), gender (17.7%), peer victimization (16.8%), and physical activity (15.9%) accounted for the largest proportions of explained variance. Similar patterns were observed across depression, anxiety, and stress subscales.

**Conclusions:**

Psychological distress among Bangladeshi adolescents is associated with the combined influence of academic pressure, peer victimization, gender‐related vulnerability, and behavioral health factors. Interventions that address educational stress and peer safety while promoting physical activity may help inform a coordinated and contextually grounded strategy for adolescent mental health prevention in Bangladesh and similar LMIC settings.

## Graphical abstract

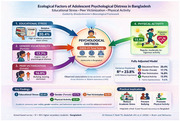

## 1 Introduction

Adolescence represents a developmental period marked by profound biological maturation, cognitive restructuring, and evolving social roles, all of which heighten vulnerability to emotional disorders (Casey et al. 2008). Globally, depression, anxiety, and stress‐related conditions constitute leading causes of morbidity among young people, with approximately one in seven adolescents experiencing a diagnosable mental health condition (World Health Organization 2025). Internalizing disorders account for a substantial proportion of disability‐adjusted life years in this age group and frequently persist into adulthood, which highlights adolescence as a critical window for prevention (Kieling et al. 2011). Although a growing body of research has examined adolescent mental health in low‐ and middle‐income countries (LMICs), much of this evidence focuses on prevalence estimates or isolated risk factors, with comparatively fewer studies adopting integrative, theory‐driven approaches (Zhang et al. 2025). In contexts such as Bangladesh, where academic competition, dense urban schooling environments, and strong familial expectations intersect (Alam and Zhu 2022; Roy et al. 2021), the ecological determinants of psychological distress may operate in distinct and contextually embedded ways. A theoretically grounded and integrative approach is therefore essential to understand how multiple domains of influence converge to shape adolescent distress.

**FIGURE 1:**
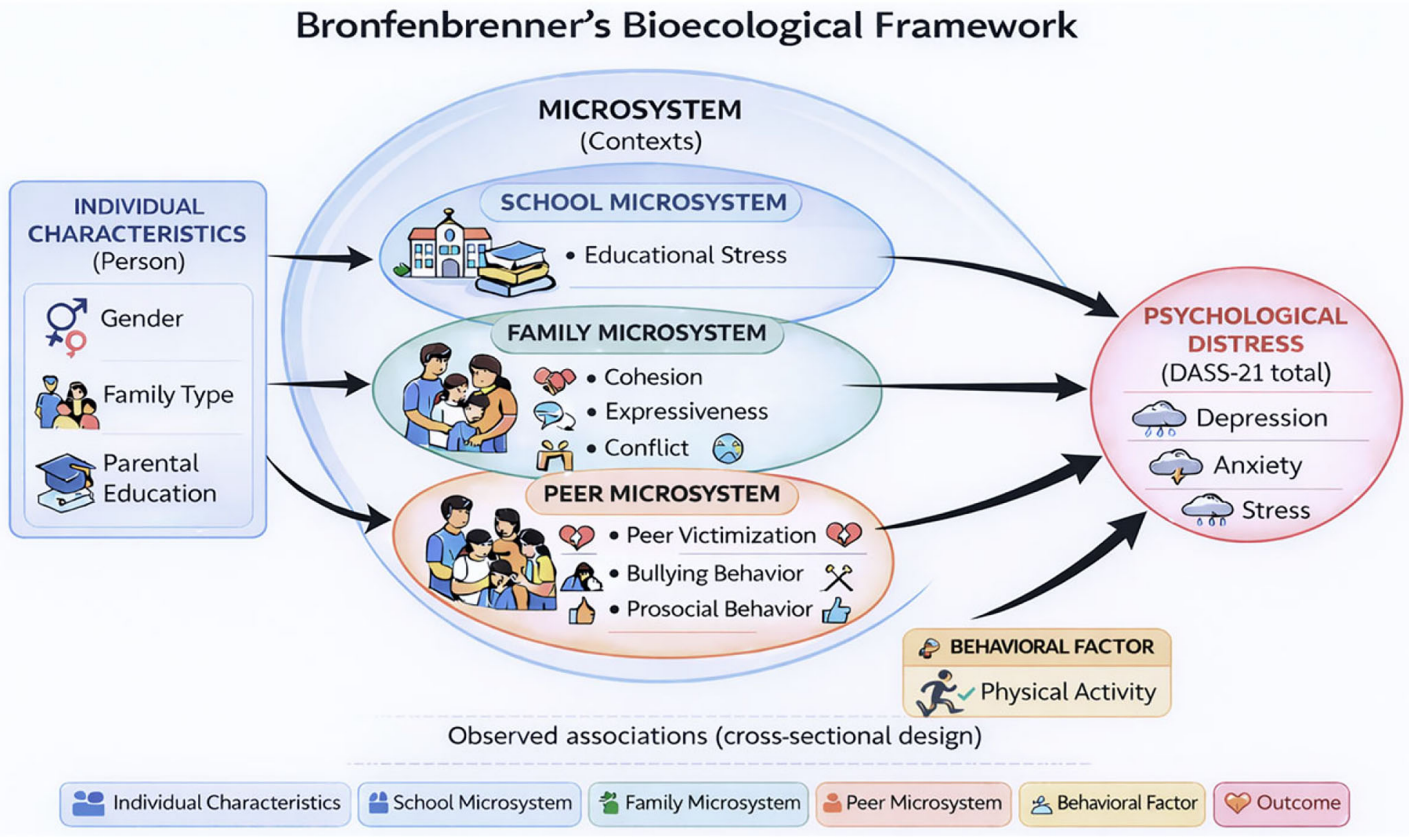
Conceptual framework based on Bronfenbrenner's bioecological framework illustrating individual, microsystem, and behavioral factors associated with adolescent psychological distress.

Bronfenbrenner's bioecological theory of human development conceptualizes development as the product of dynamic interactions between individuals and their nested environmental systems (Bronfenbrenner and Morris 2007). The model distinguishes multiple layers of influence, including the microsystem (immediate environments such as family, school, and peers), with proximal processes—recurring interactions within these contexts—considered the primary drivers of developmental outcomes (Tudge et al. 2009). Within this framework, proximal processes occurring in adolescents’ immediate contexts—particularly schools, families, and peer networks—are central to psychological outcomes. In the present study, we focus on microsystem‐level factors that are most proximal to adolescents’ daily experiences, including educational stress (school), peer victimization (peer), and family relationship dimensions (family). Physical activity is considered a behavioral factor embedded within adolescents’ daily contexts, whereas demographic characteristics are treated as individual‐level attributes. A schematic representation of the conceptual framework is presented in Figure 1. During adolescence, the school environment becomes especially salient as academic demands intensify and performance evaluations carry long‐term educational implications. Empirical evidence consistently links academic pressure and educational stress with elevated depressive and anxiety symptoms (Deb et al. 2015; Pascoe et al. 2020; Steare et al. 2023). In Bangladesh, these pressures may be amplified by competitive examination systems and sociocultural norms emphasizing academic achievement as a primary pathway to social mobility (Alam and Zhu 2022; Al‐Mamun, Mamun, et al. 2024). Within such high‐stakes environments, sustained academic stress may function as a persistent psychosocial strain embedded within broader structural expectations (Chyu and Chen 2022; Lee and Larson 2000).

Family processes constitute another core microsystem shaping adolescent emotional functioning. Cohesive and communicative family environments are associated with lower levels of psychological distress, whereas conflictual or poorly functioning family relationships are consistently linked to internalizing and externalizing difficulties (Demetriou 2025; Feng et al. 2025; Oltean et al. 2020; Zhang et al. 2024). Supportive family dynamics may foster emotional regulation and adaptive coping by providing validation, guidance, and relational security, whereas persistent conflict may undermine perceived safety and heighten vulnerability to stress (Lunkenheimer et al. 2007). In contrast, environments characterized by low cohesion or limited expressiveness may restrict adolescents’ opportunities to process and manage stress effectively. At the same time, peer relationships assume heightened developmental salience during adolescence as identity formation increasingly unfolds within social networks. Experiences of peer victimization have demonstrated robust and longitudinal associations with depression, anxiety, and stress across diverse cultural contexts (Arseneault 2017; Zhu et al. 2024). Victimization may function as a chronic interpersonal stressor that disrupts belongingness and increases emotional vulnerability (Cheng et al. 2025). Within the bioecological framework, such peer experiences represent proximal processes within the peer microsystem that may contribute to psychological distress. Within school‐centered social ecologies, peer victimization may compound existing stress exposures by reducing perceived social support and increasing sensitivity to social threat (Salmivalli 2010; van Harmelen et al. 2016).

Although psychosocial stressors are central to understanding adolescent distress, behavioral health factors such as physical activity represent potentially modifiable correlates within the same ecological framework. A growing body of evidence links higher levels of physical activity to lower depressive and anxiety symptoms among adolescents (Wanjau et al. 2023). The stress‐buffering hypothesis suggests that physical activity may mitigate the psychological impact of stress through mechanisms such as improved affect regulation, enhanced stress response modulation, better sleep quality, and strengthened self‐efficacy (Cohen and Wills 1985; Lubans et al. 2016). Although neurobiological pathways—including regulation of the hypothalamic–pituitary–adrenal (HPA) axis and adaptive changes in neural functioning—have been proposed as underlying mechanisms (Stillman et al. 2016; Zschucke et al. 2013), much of the existing literature has examined physical activity independently rather than situating it within comprehensive models that simultaneously account for academic, familial, and peer stressors (Biddle and Asare 2011). Within the bioecological framework, physical activity can be conceptualized as a behavioral factor embedded within adolescents’ daily contexts that may interact with proximal processes across multiple microsystems. Consequently, it remains unclear whether physical activity retains an independent association with psychological distress after adjustment for salient psychosocial exposures, particularly in LMIC settings where sedentary behavior is prevalent and contextual stressors may be pronounced (Guthold et al. 2020).

In Bangladesh, research on adolescent mental health has expanded in recent years; however, most studies have examined isolated risk factors rather than simultaneously modeling multiple ecological domains (Al Mamun et al. 2021; Al‐Mamun, Islam, et al. 2024; Anjum et al. 2022; Ria et al. 2022). Previous investigations have largely focused on prevalence estimates or single domains of risk such as depressive symptoms or anxiety without embedding findings within comprehensive ecological frameworks (Anjum et al. 2022; Ria et al. 2022). Although recent ecological analyses in Bangladesh have highlighted the importance of microsystem influences—including family, school, and peer contexts—in shaping psychological outcomes (Ahmed and Saha 2023; Arafat and Saleem 2025), there remain very limited studies that apply a theoretically structured approach integrating demographic, educational, family, peer, and behavioral factors within a single analytical model among adolescents. Moreover, limited evidence exists regarding the relative contribution of these domains to overall psychological distress and to specific symptom clusters (Ahmed et al. 2023). Quantifying the relative importance of correlated factors is therefore essential, as conventional regression coefficients may not accurately represent their proportional contribution within complex social ecologies (Johnson and LeBreton 2004). Understanding not only whether these factors are associated with distress but also how much they contribute relative to one another is critical for prioritizing intervention targets and informing evidence‐based policy in resource‐limited settings (Patel et al. 2018).

Guided by Bronfenbrenner's bioecological theory of human development and the stress‐buffering framework, the present study examined psychological distress among Bangladeshi adolescents within an integrated ecological context. Specifically, we investigated the relative contributions of demographic characteristics, educational stress, family relationship dimensions, peer relationship factors, and physical activity in relation to overall psychological distress. Hierarchical regression models were used to reflect theoretically grouped ecological domains and to evaluate the incremental explanatory power (Δ*R* ^2^) of successive factor blocks. Importantly, the ordering of variables reflects theoretical organization based on the bioecological framework rather than temporal or causal sequencing. Relative importance analysis was further applied to estimate each factor's proportional contribution to explained variance. Domain‐specific regression analyses were additionally conducted to determine whether patterns of association differed across depression, anxiety, and stress symptoms. By integrating psychosocial and behavioral determinants within a coherent ecological framework, this study advances understanding of contextually salient and potentially modifiable factors associated with adolescent psychological distress in Bangladesh.

## 2 Methods

### 2.1 Study Design, Setting, and Participants

This school‐based cross‐sectional study was conducted among higher secondary students in Dhaka, Bangladesh, between May and August 2025. Data were collected from two large government‐affiliated colleges, Birshreshtha Noor Mohammad Public College and Birshreshtha Munshi Abdur Rouf Public College, which serve students from diverse socioeconomic and academic backgrounds. These institutions represent established public colleges in the city and enroll students from varied academic streams.

Participants were recruited using a non‐probability convenience sampling approach. Students were considered eligible if they were officially enrolled in the selected institutions, present during the survey period, and willing to participate. All eligible students who were present on the days of data collection were invited to take part in the study. Questionnaires with missing responses on primary outcome measures were excluded prior to statistical analysis.

Data were collected using a structured, self‐administered questionnaire distributed in printed format during regular classroom sessions. Prior to initiating data collection, the research team met with college authorities and obtained formal administrative approval from institutional heads. Following approval, students were approached during scheduled class hours and provided with a standardized explanation of the study's objectives, procedures, and voluntary nature. Completion of the questionnaire required approximately 30–40 min.

Participation was entirely voluntary, and students were informed that refusal or withdrawal would not affect their academic standing or personal circumstances. Written informed consent was obtained from all participants before survey administration, and assent procedures were implemented for students under 18 years of age in accordance with approved ethical guidelines. To ensure confidentiality, no personally identifiable information was collected, and responses were recorded anonymously. Immediately after collection, questionnaires were reviewed for completeness to minimize missing data. A total of 503 questionnaires were returned; two were excluded due to incomplete primary outcome data, resulting in a final analytical sample of 501 adolescents.

### 2.2 Measures

All instruments were administered in Bangla (Bengali), the native language of participants. The Depression Anxiety and Stress Scale 21 (DASS‐21) was used in its previously validated Bangla version. For the remaining instruments, translation was conducted following standard forward–backward translation procedures (Beaton et al. 2000). The original English versions were translated into Bangla by bilingual researchers and independently back‐translated into English by a separate translator. Discrepancies were reviewed and resolved to ensure semantic and conceptual equivalence. Internal consistency of all scales was assessed within the present sample.

#### 2.2.1 Sociodemographic Information

Participants provided information on key demographic variables, including gender (male or female), type of family structure (nuclear or joint), and parental educational background. Parental education was grouped into three categories: up to secondary level, higher secondary level, and bachelor's degree or higher.

#### 2.2.2 Physical Activity

Engagement in physical activity was measured using the physical activity domain of the Adolescent Health Promotion (AHP) scale (Chen et al. 2003). This domain includes five items assessing the frequency of health‐promoting physical activity behaviors. Each item is rated on a 5‐point response scale from 1 (never) to 5 (always). Composite scores range from 5 to 25, with higher values indicating more frequent participation in physical activity. The internal consistency of this subscale was good in the present study (Cronbach's *α* = 0.81).

#### 2.2.3 Educational Stress

Perceived academic stress was evaluated using the Educational Stress Scale for Adolescents (ESSA) (Sun et al. 2011). The ESSA contains 16 items capturing perceived pressure related to academic expectations, workload, and school‐related demands. Items are rated on a 5‐point Likert scale from 1 (strongly disagree) to 5 (strongly agree). Total scores can range from 16 to 80, with higher scores representing greater levels of educational stress. The scale showed strong internal consistency in this sample (Cronbach's *α* = 0.87).

#### 2.2.4 Family Relationship

Family relational functioning was assessed using the Brief Family Relationship Scale (BFRS) (Fok et al. 2014). This instrument evaluates three dimensions of family interaction: cohesion, expressiveness, and conflict. The 16 items are rated on a 3‐point scale (1 = “not at all,” 2 = “somewhat,” 3 = “a lot”). Items pertaining to family conflict were reverse‐coded prior to computing total scores. Higher overall scores indicate more positive family relationship quality. The scale demonstrated high reliability in the present sample (Cronbach's *α* = 0.89).

#### 2.2.5 Peer Relationship

Peer interaction patterns were measured using the Peer Relationship Questionnaire (PRQ) (Rigby and Slee 1993). The PRQ assesses three aspects of peer dynamics: bullying behavior, experiences of victimization, and prosocial behavior. Items are scored on a 4‐point scale ranging from 1 (never) to 4 (very often). Aggregate scores range from 12 to 48, with higher scores reflecting greater frequency of the respective behaviors. Internal consistency in the present sample was acceptable (Cronbach's *α* = 0.73).

#### 2.2.6 Psychological Distress

Symptoms of psychological distress were assessed using the 21‐item DASS‐21 (Lovibond 1995). The scale consists of three seven‐item subscales designed to capture depressive symptoms, anxiety symptoms, and stress‐related experiences. Responses are recorded on a 4‐point scale ranging from 0 (did not apply to me at all) to 3 (applied to me very much or most of the time). Following standard scoring procedures, subscale totals were summed and multiplied by two to obtain severity scores comparable to the full DASS version, resulting in a possible total score range of 0–126. Higher scores reflect greater overall psychological distress. In the current sample, the scale demonstrated excellent internal reliability (Cronbach's *α* = 0.97).

### 2.3 Statistical Analysis

All statistical analyses were conducted using R version 4.4.1. Participant characteristics were summarized using means and standard deviations (SDs) for continuous variables and frequencies with percentages for categorical variables.

Pearson correlation coefficients were computed to examine bivariate associations among continuous study variables, including DASS‐21 subscales (depression, anxiety, and stress), physical activity, educational stress, family relationship dimensions (cohesion, expressiveness, and conflict), and peer relationship indicators (bullying behavior, peer victimization, and prosocial behavior). Correlation matrices included corresponding *p* values, with statistical significance evaluated at conventional thresholds (*p* < 0.05, *p* < 0.01, and *p* < 0.001).

To examine factor associated with overall psychological distress (DASS‐21 total score), hierarchical multiple linear regression analyses were performed using four sequential models reflecting the theoretically grouped ecological domains. Model 1 included demographic characteristics (gender, family type, father's education, and mother's education), where Model 2 added educational stress. Model 3 further incorporated family relationship variables (cohesion, expressiveness, and conflict) and peer relationship variables (bullying behavior, peer victimization, and prosocial behavior). Model 4 (final model) additionally included physical activity. Variable selection and block ordering were guided by the bioecological framework and stress‐buffering theory. Importantly, the ordering of variables reflects theoretical organization rather than temporal or causal sequencing. Model fit was assessed using *R* ^2^, adjusted *R* ^2^, and overall *F*‐statistics. Incremental contributions of each block were quantified using changes in explained variance (Δ*R* ^2^) across successive models. Regression coefficients (*B*) were reported with heteroskedasticity‐consistent (HC3) robust standard errors, 95% confidence intervals (CIs), and two‐tailed *p* values.

Model assumptions were evaluated prior to interpretation. Heteroskedasticity was assessed using the Breusch–Pagan test. Because evidence of heteroskedasticity was observed in models including psychosocial factors, statistical inference was based on HC3 robust standard errors, which provide more reliable estimates under heteroskedasticity and are recommended for moderate sample sizes. Multicollinearity was evaluated using variance inflation factors (VIFs), with no indication of problematic collinearity detected.

To quantify the relative contribution of each factor in the final psychological distress model, relative importance analysis was conducted using the Lindeman–Merenda–Gold (LMG) method. The LMG metric decomposes total model *R* ^2^ into the average incremental contribution of each factor across all possible orderings of factors, thereby estimating each variable's proportional contribution to explained variance. Results are presented as percentages summing to 100%.

Finally, to examine whether factor demonstrated consistent patterns across symptom domains, three additional multiple linear regression models were fitted using the same covariate set as the final distress model, with DASS‐21 depression, anxiety, and stress specified as separate outcomes. For each subscale model, regression coefficients (*B*) with HC3 robust standard errors, 95% CIs, and *p* values were reported alongside model fit indices (*R* ^2^, adjusted *R* ^2^, and overall *F*‐test statistics). A two‐tailed *p* value <0.05 was considered statistically significant.

### 2.4 Artificial Intelligence Disclosure

Generative AI tools (ChatGPT and OpenAI) were used for language refinement and editing of the manuscript. The authors reviewed and approved all content and take full responsibility for the integrity and accuracy of the manuscript.

## 3 Results

### 3.1 Characteristics of the Participants

A total of 501 adolescents were included in the final analysis. The majority were female (67.5%) and from nuclear families (91.4%). Over half of the fathers (56.7%) and nearly half of the mothers (49.1%) had attained a bachelor's degree or higher (Table 1).

**TABLE 1:** Sociodemographic characteristics and descriptive statistics of study variables (*N* = 501).

Characteristic	*N* (%) or mean (SD)
Gender	
Male	163 (32.5%)
Female	338 (67.5%)
Family type	
Nuclear	458 (91.4%)
Joint	43 (8.6%)
Father's education	
Up to secondary	72 (14.4%)
Higher secondary	145 (28.9%)
Bachelor or above	284 (56.7%)
Mother's education	
Up to secondary	98 (19.6%)
Higher secondary	157 (31.3%)
Bachelor or above	246 (49.1%)
Psychological distress	47.19 (31.83)
Depression	16.26 (11.02)
Anxiety	15.64 (10.97)
Stress	15.29 (10.93)
Physical activity	13.80 (4.91)
Educational stress	55.91 (10.96)
Family relationship	
Cohesion	17.16 (3.09)
Expressiveness	6.89 (1.72)
Conflict	14.87 (3.10)
Peer relationship	
Bullying behavior	4.86 (1.67)
Peer victimization	6.30 (2.69)
Prosocial	12.01 (2.99)

Abbreviation: SD, standard deviation.

The mean total psychological distress score (DASS‐21) was 47.19 (SD = 31.83). Mean subscale scores were 16.26 (SD = 11.02) for depression, 15.64 (SD = 10.97) for anxiety, and 15.29 (SD = 10.93) for stress. The mean physical activity score was 13.80 (SD = 4.91), whereas the mean educational stress score was 55.91 (SD = 10.96). Family relationship scores indicated mean values of 17.16 (SD = 3.09) for cohesion, 6.89 (SD = 1.72) for expressiveness, and 14.87 (SD = 3.10) for conflict. For peer relationship domains, mean scores were 4.86 (SD = 1.67) for bullying behavior, 6.30 (SD = 2.69) for peer victimization, and 12.01 (SD = 2.99) for prosocial behavior.

### 3.2 Bivariate Associations Among Study Variables

Depression, anxiety, and stress were strongly intercorrelated (*r* = 0.895–0.911, all *p* < 0.001), indicating substantial overlap among symptom domains. Physical activity was significantly and inversely associated with depression (*r* = −0.252), anxiety (*r* = −0.275), and stress (*r* = −0.239) (all *p *< 0.001). Educational stress demonstrated positive associations with all three subscales (*r* = 0.241–0.264, all *p* < 0.001). Family cohesion and expressiveness were negatively correlated with psychological symptoms (all *p *< 0.001), whereas family conflict showed weaker but statistically significant positive associations. Peer victimization was positively associated with depression (*r* = 0.222), anxiety (*r* = 0.257), and stress (*r* = 0.269) (all *p* < 0.001). Prosocial behavior showed small positive correlations with psychological outcomes, and bullying behavior was weakly but significantly associated with higher distress levels (Table 2).

**TABLE 2:** Pearson correlation matrix among study variables.

Variable	1	2	3	4	5	6	7	8	9	10	11
1. Depression	1.000										
2. Anxiety	0.895***	1.000									
3. Stress	0.902***	0.911***	1.000								
4. Physical activity	−0.252***	−0.275***	−0.239***	1.000							
5. Educational stress	0.241***	0.264***	0.264***	−0.113*	1.000						
6. Family cohesion	−0.170***	−0.205***	−0.207***	0.146**	0.033	1.000					
7. Family expressiveness	−0.200***	−0.228***	−0.234***	0.193***	−0.057	0.677***	1.000				
8. Family conflict	−0.092*	−0.112*	−0.123**	0.020	−0.000	0.411***	0.279***	1.000			
9. Bullying behavior	0.090*	0.098*	0.098*	−0.016	0.051	−0.138**	−0.063	−0.129**	1.000		
10. Peer victimization	0.222***	0.257***	0.269***	−0.037	0.168***	−0.173***	−0.195***	−0.180***	0.387***	1.000	
11. Prosocial behavior	0.140**	0.128**	0.127**	−0.088	0.119**	0.161***	0.070	0.136**	−0.074	0.141**	1.000

*** *p* <0.001

** *p* <0.001

* *p* <0.05

### 3.3 Hierarchical Regression Analyses Predicting Psychological Distress

In Model 1, demographic characteristics accounted for 6.5% of the variance in psychological distress (*R* ^2^ = 0.065). Female adolescents reported significantly higher distress compared with males (*B* = 16.19, *p* < 0.001). Adding educational stress in Model 2 significantly improved model fit (Δ*R* ^2^ = 0.059), with educational stress showing a significant positive association with psychological distress (*B* = 0.73, *p* < 0.001). Model 3 incorporated family and peer relationship variables, resulting in a further increase in explained variance (Δ*R* ^2^ = 0.097; total *R* ^2^ = 0.221). In this model, peer victimization (*B* = 2.05, *p* < 0.001) and family expressiveness (*B* = −2.11, *p* = 0.041) were significantly associated with psychological distress. In the final model (Model 4), the inclusion of physical activity led to an additional improvement in model fit (Δ*R* ^2^ = 0.017), with physical activity independently associated with lower psychological distress (*B* = −0.91, 95% CI [−1.53, −0.29], *p* = 0.004). The full model explained 23.8% of the variance in psychological distress (*R* ^2^ = 0.238). Female gender, educational stress, peer victimization, and physical activity remained significantly associated with psychological distress in the fully adjusted model (Table 3).

**TABLE 3:** Hierarchical multiple linear regression predicting overall psychological distress (Depression Anxiety and Stress Scale 21 [DASS‐21] total score).

Factor	Model 1			Model 2			Model 3			Model 4		
	*B* (SE)	95% CI	*p*	*B* (SE)	95% CI	*p*	*B* (SE)	95% CI	*p*	B (SE)	95% CI	*p*
Intercept	37.00 (4.42)	[28.32, 45.68]	<0.001	0.89 (8.45)	[−15.71, 17.49]	0.916	18.02 (15.13)	[−11.72, 47.75]	0.234	30.86 (16.55)	[−1.66, 63.38]	0.063
Female (vs. male)	16.19 (2.89)	[10.50, 21.87]	<0.001	13.30 (2.97)	[7.45, 19.14]	<0.001	15.13 (3.05)	[9.14, 21.11]	<0.001	12.32 (3.14)	[6.15, 18.49]	<0.001
Joint family (vs. nuclear)	9.58 (5.16)	[−0.56, 19.73]	0.064	12.07 (5.34)	[1.59, 22.56]	0.024	10.31 (5.09)	[0.30, 20.32]	0.043	9.61 (4.93)	[−0.07, 19.30]	0.052
Father education (higher secondary)	−2.46 (5.31)	[−12.90, 7.98]	0.644	−4.05 (5.12)	[−14.11, 6.01]	0.429	−1.92 (4.77)	[−11.29, 7.46]	0.688	−1.49 (4.80)	[−10.91, 7.94]	0.757
Father education (bachelor or above)	−3.52 (6.10)	[−15.51, 8.46]	0.564	−6.68 (5.76)	[−18.00, 4.64]	0.247	−4.69 (5.29)	[−15.08, 5.70]	0.376	−4.20 (5.32)	[−14.66, 6.25]	0.430
Mother education (higher secondary)	0.14 (4.72)	[−9.13, 9.40]	0.977	−1.64 (4.70)	[−10.88, 7.60]	0.728	−0.21 (4.26)	[−8.57, 8.16]	0.961	−0.66 (4.24)	[−8.99, 7.68]	0.877
Mother education (bachelor or above)	2.26 (5.58)	[−8.69, 13.21]	0.686	1.91 (5.36)	[−8.63, 12.44]	0.722	1.99 (4.90)	[−7.63, 11.61]	0.685	1.83 (4.90)	[−7.79, 11.46]	.709
Educational stress	—	—	—	0.73 (0.15)	[0.43, 1.03]	<0.001	0.58 (0.14)	[0.30, 0.86]	<0.001	0.56 (0.14)	[0.29, 0.83]	<0.001
Family cohesion	—	—	—	—	—	—	−1.13 (0.62)	[−2.35, 0.08]	0.067	−1.03 (0.62)	[−2.24, 0.19]	0.097
Family expressiveness	—	—	—	—	—	—	−2.11 (1.03)	[−4.14, −0.09]	0.041	−1.67 (1.05)	[−3.73, 0.39]	0.111
Family conflict	—	—	—	—	—	—	−0.16 (0.47)	[−1.08, 0.76]	0.737	−0.22 (0.46)	[−1.12, 0.68]	0.632
Bullying behavior	—	—	—	—	—	—	0.61 (1.13)	[−1.62, 2.83]	0.591	0.48 (1.18)	[−1.85, 2.80]	0.687
Peer victimization	—	—	—	—	—	—	2.05 (0.59)	[0.89, 3.20]	<0.001	2.07 (0.58)	[0.93, 3.22]	<0.001
Prosocial behavior	—	—	—	—	—	—	0.70 (0.46)	[−0.21, 1.60]	0.132	0.64 (0.46)	[−0.25, 1.54]	0.158
Physical activity	—	—	—	—	—	—	—	—	—	−0.91 (0.32)	[−1.53, −0.29]	0.004
Model fit	*R* ^2^ = 0.065; adjusted *R* ^2^ = 0.054; *F* (6,494) = 5.74; *p* < 0.001			*R* ^2^ = 0.125; adjusted *R* ^2^ = 0.112; Δ*R* ^2^ = 0.059; *F* (7,493) = 10.01; *p* < 0.001			*R* ^2^ = 0.221; adjusted *R* ^2^ = 0.201; Δ*R* ^2^ = 0.097; *F* (13,487) = 10.65; *p* < 0.001			*R* ^2^ = 0.238; adjusted *R* ^2^ = 0.216; Δ*R* ^2^ = 0.017; *F* (14,486) = 10.87; *p* < 0.001		

*Note: B*, regression coefficient; SE, HC3 robust standard error; 95% CI, 95% confidence interval.

### 3.4 Relative Importance of Factors of Psychological Distress

Relative importance analysis using the LMG metric indicated that educational stress accounted for the largest proportion of explained variance (20.4%), followed by gender (17.7%), peer victimization (16.8%), and physical activity (15.9%). Family expressiveness (9.6%) and family cohesion (7.8%) contributed moderate proportions, whereas parental education and family conflict accounted for minimal variance (Table 4).

**TABLE 4:** Relative importance of factors in the final psychological distress model (Lindeman–Merenda–Gold [LMG] decomposition).

Factor	Relative importance (%)
Educational stress	20.4
Gender	17.7
Peer victimization	16.8
Physical activity	15.9
Family expressiveness	9.6
Family cohesion	7.8
Prosocial behavior	4.4
Family type	2.7
Bullying behavior	1.9
Family conflict	1.9
Father education	0.4
Mother education	0.4

### 3.5 Regression Analyses for Depression, Anxiety, and Stress Subscales

Across fully adjusted models examining depression, anxiety, and stress separately, female gender, educational stress, and peer victimization were consistently associated with higher symptom levels, whereas physical activity was associated with lower symptom levels. Specifically, female gender was positively associated with depression (*B* = 3.89, *p* < 0.001), anxiety (*B* = 4.71, *p* < 0.001), and stress (*B* = 3.72, *p* < 0.001). Educational stress was positively associated with depression (*B* = 0.17, *p* < 0.001), anxiety (*B* = 0.19, *p* < 0.001), and stress (*B* = 0.20, *p* < 0.001). Peer victimization remained a significant positive factor across all three outcomes. Physical activity demonstrated significant inverse associations with depression (*B* = −0.32, *p* = 0.004), anxiety (*B* = −0.33, *p* = 0.003), and stress (*B* = −0.27, *p* = 0.012). Among the three symptom domains, the anxiety model explained the greatest proportion of variance (*R* ^2^ = 0.252), followed by stress (*R* ^2^ = 0.227) and depression (*R* ^2^ = 0.197) (Table 5).

**TABLE 5:** Multiple linear regression models predicting depression, anxiety, and stress subscales.

Factors	Depression			Anxiety			Stress		
	*B* (SE)	95% CI	*p*	*B* (SE)	95% CI	*p*	*B* (SE)	95% CI	*p*
Intercept	9.89 (5.82)	[−1.54, 21.33]	0.090	10.54 (5.48)	[−0.23, 21.31]	0.055	10.43 (5.64)	[−0.65, 21.51]	0.065
Female (vs. male)	3.89 (1.11)	[1.71, 6.06]	<0.001	4.71 (1.06)	[2.62, 6.80]	<0.001	3.72 (1.07)	[1.63, 5.82]	<0.001
Joint family (vs. nuclear)	2.99 (1.63)	[−0.22, 6.20]	0.068	3.93 (1.83)	[0.34, 7.52]	0.032	2.69 (1.69)	[−0.64, 6.02]	0.113
Father education (higher secondary)	0.23 (1.66)	[−3.03, 3.48]	0.890	−0.48 (1.67)	[−3.76, 2.80]	0.774	−1.24 (1.67)	[−4.51, 2.04]	0.458
Father education (bachelor or above)	−0.55 (1.84)	[−4.16, 3.07]	0.766	−1.23 (1.86)	[−4.87, 2.42]	0.509	−2.43 (1.89)	[−6.15, 1.28]	0.199
Mother education (higher secondary)	0.31 (1.49)	[−2.62, 3.24]	0.835	−0.93 (1.47)	[−3.82, 1.95]	0.526	−0.03 (1.46)	[−2.91, 2.84]	0.982
Mother education (bachelor or above)	0.80 (1.74)	[−2.62, 4.21]	0.647	−0.15 (1.69)	[−3.48, 3.17]	0.928	1.19 (1.72)	[−2.18, 4.56]	0.489
Educational stress	0.17 (0.05)	[0.07, 0.26]	<0.001	0.19 (0.05)	[0.10, 0.28]	<0.001	0.20 (0.05)	[0.10, 0.29]	<0.001
Family cohesion	−0.28 (0.22)	[−0.71, 0.14]	0.192	−0.37 (0.21)	[−0.78, 0.04]	0.074	−0.37 (0.21)	[−0.79, 0.06]	0.088
Family expressiveness	−0.56 (0.38)	[−1.30, 0.18]	0.139	−0.54 (0.36)	[−1.24, 0.17]	0.134	−0.58 (0.37)	[−1.29, 0.14]	0.115
Family conflict	−0.06 (0.16)	[−0.38, 0.26]	0.703	−0.06 (0.15)	[−0.36, 0.24]	0.708	−0.10 (0.16)	[−0.42, 0.22]	0.534
Bullying behavior	0.20 (0.41)	[−0.60, 0.99]	0.631	0.17 (0.39)	[−0.61, 0.94]	0.668	0.11 (0.40)	[−0.68, 0.90]	0.781
Peer victimization	0.59 (0.20)	[0.19, 0.99]	0.004	0.73 (0.20)	[0.34, 1.11]	<0.001	0.75 (0.20)	[0.35, 1.16]	<0.001
Prosocial behavior	0.27 (0.16)	[−0.04, 0.58]	0.093	0.17 (0.16)	[−0.13, 0.48]	0.264	0.20 (0.16)	[−0.11, 0.52]	0.198
Physical activity	−0.32 (0.11)	[−0.53, −0.10]	0.004	−0.33 (0.11)	[−0.54, −0.11]	0.003	−0.27 (0.11)	[−0.48, −0.06]	0.012
Model fit	*R* ^2^ = 0.197; adjusted *R* ^2^ = 0.174; *F* (14,486) = 8.50; *p* < 0.001			*R* ^2^ = 0.252; adjusted *R* ^2^ = 0.230; *F* (14,486) = 11.68; *p* < 0.001			*R* ^2^ = 0.227; adjusted *R* ^2^ = 0.205; *F* (14,486) = 10.22; *p* < 0.001		

*Note*: B, regression coefficient; SE, HC3 robust standard error; 95% CI, 95% confidence interval. All models adjusted for demographic, educational stress, family, peer, and physical activity variables.

## 4 Discussion

The present study examined psychological distress among Bangladeshi adolescents through the lens of Bronfenbrenner's bioecological theory, integrating educational, familial, peer, and behavioral domains within a unified analytical framework. The findings indicate that adolescent distress is associated not with isolated risk factors but with co‐occurring influences embedded within daily microsystems. Educational stress emerged as the most prominent correlate, whereas gender and peer victimization demonstrated consistent positive associations across psychological symptom domains. In contrast, physical activity was inversely associated with psychological distress, retaining significance even after adjustment for psychosocial exposures. These findings highlight the interplay between contextual stressors and modifiable behavioral factors associated with adolescent emotional well‐being.

Female adolescents reported significantly higher levels of overall psychological distress as well as elevated depression, anxiety, and stress symptoms. This pattern is consistent with robust international evidence indicating that internalizing symptoms increase during adolescence and are more prevalent among girls, particularly for depression and anxiety (Campbell et al. 2021; Matud et al. 2024; Yoon et al. 2022). Pubertal transitions, gendered stress exposures, and differences in coping styles and emotion regulation have been proposed as potential mechanisms associated with this disparity. The World Health Organization identifies adolescence as a critical period for mental health vulnerability, with global estimates underscoring the substantial burden of mental health conditions in this age group (World Health Organization 2025). Within Bangladesh, emerging school‐based research likewise documents considerable psychological symptomatology among adolescents (Al‐Mamun, Islam, et al. 2024), suggesting that gender disparities in distress are not confined to Western contexts but are also evident within South Asian sociocultural settings.

Educational stress emerged as a robust and consistent factor associated with overall psychological distress as well as each DASS‐21 subscale, and it accounted for the largest share of explained variance in the relative importance analysis. This finding reinforces the central role of the school microsystem within Bronfenbrenner's bioecological framework, where daily proximal processes—such as examinations, performance evaluations, and competitive academic comparison—structure adolescents’ lived experiences. In Bangladesh, where educational attainment is closely tied to social mobility and familial expectations, academic demands may extend beyond routine challenges to become sustained psychosocial pressures embedded within broader structural aspirations. Consistent with international evidence linking academic stress to poorer psychological well‐being (Pascoe et al. 2020; Steare et al. 2023), the present results suggest that persistent educational stress may be linked to emotional functioning across depression, anxiety, and stress domains. Bangladesh‐specific findings similarly document substantial stress symptoms among school‐going adolescents (Roy et al. 2021), further supporting the interpretation that educational stress represents a contextually grounded and developmentally salient factor associated with adolescent psychological distress.

Peer victimization was consistently associated with higher overall psychological distress as well as elevated depression, anxiety, and stress symptoms. Within the bioecological framework outlined in the Introduction, peer relationships constitute a central component of the adolescent microsystem, particularly in school‐centered social environments where identity formation and social status are developmentally salient. In such contexts, experiences of victimization may function as chronic interpersonal stressors, heightening perceptions of social threat, humiliation, and diminished belonging (Ajibewa et al. 2025; Schacter et al. 2025). This interpretation aligns with robust international evidence linking peer victimization to internalizing difficulties across diverse cultural settings (Arseneault 2017; Zhu et al. 2024). Notably, bullying perpetration did not retain a significant association with distress in adjusted models, whereas victimization demonstrated a stable and robust relationship, suggesting that being targeted—rather than engaging in aggressive behavior—may be more closely associated with internalizing symptom burden (Eastman et al. 2018). Consistent with the ecological perspective advanced earlier, these findings indicate that peer safety within the school microsystem represents a key contextual factor associated with adolescent emotional well‐being.

Physical activity was inversely associated with overall psychological distress as well as each symptom domain, and this relationship persisted after adjustment for demographic characteristics, educational stress, family dynamics, and peer experiences. Within the ecological framework outlined earlier, physical activity may function as a behavioral resource embedded in adolescents’ daily contexts, offering regulatory and restorative benefits amid psychosocial strain. This finding is consistent with a substantial body of evidence linking higher levels of physical activity to better mental health outcomes among children and adolescents, including lower depressive and anxiety symptoms (Biddle and Asare 2011; Lubans et al. 2016; Wanjau et al. 2023). From a stress‐buffering perspective (Cohen and Wills 1985), regular physical activity may enhance affect regulation, improve sleep and physiological stress responses, and strengthen self‐efficacy and social connectedness (Korkutata et al. 2025; Tikac et al. 2022). Notably, the absence of a significant interaction between physical activity and educational stress indicates that its association with distress in this study was additive rather than moderating. In other words, higher physical activity was linked to lower distress overall, independent of academic strain and peer victimization, highlighting its potential role as a broadly beneficial behavioral correlate within adolescent mental health promotion.

The LMG variance decomposition extends the hierarchical regression findings by estimating each factor's proportional contribution to explained variance across all possible model orderings. In the present study, educational stress, gender, peer victimization, and physical activity accounted for the largest shares of variance in psychological distress, indicating their relative prominence within the multivariable ecological model. This analytic approach strengthens the interpretive weight of the findings by moving beyond statistical significance to clarify comparative influence. Collectively, the results highlight school‐related stressors and peer safety as important considerations for intervention prioritization, while also identifying physical activity as a modifiable behavioral correlate that retains independent relevance within adolescents’ broader ecological contexts.

### 4.1 Implications of the Findings

The present findings carry meaningful implications for adolescent mental health promotion in Bangladesh and comparable LMIC contexts. The prominence of educational stress underscores the central role of the school environment as a strategic site for preventive intervention. Given its substantial relative contribution to psychological distress, school‐based initiatives that address academic pressure—such as structured stress‐management programs, examination preparation support, academic counseling services, and teacher training in student mental health awareness—may help address chronic academic strain. Reducing performance‐related stress within high‐stakes educational systems may therefore represent a critical leverage point for population‐level prevention. The consistent association between peer victimization and distress further highlights the importance of fostering safe and supportive peer climates within educational institutions. Comprehensive anti‐bullying policies, school climate interventions, and programs that promote inclusivity and belongingness may contribute to reducing the emotional burden associated with victimization. Strengthening peer norms and enhancing students’ sense of social safety may be particularly relevant during adolescence, when peer relationships become developmentally salient. Finally, the inverse association between physical activity and psychological distress suggests that behavioral health promotion should be integrated into mental health strategies. Incorporating structured physical activity into school routines—through strengthened physical education curricula, extracurricular sports programs, and community‐based youth activity initiatives—offers a feasible and potentially scalable approach with both physiological and psychosocial benefits.

### 4.2 Strengths, Limitations, and Future Directions

This study offers several important contributions. By applying a theoretically grounded ecological framework, it integrated demographic, educational, family, peer, and behavioral domains within a single analytic model, moving beyond the single‐risk‐factor approaches that characterize much of the regional literature. The hierarchical modeling strategy enabled evaluation of the incremental influence of theoretically ordered factor blocks, whereas the use of relative importance analysis provided a nuanced estimate of each factor's proportional contribution to explained variance—an analytic approach rarely applied in adolescent mental health research in Bangladesh. The examination of both overall psychological distress and domain‐specific symptom clusters further strengthens the robustness of the findings, demonstrating consistent patterns across depression, anxiety, and stress. Additionally, the use of heteroskedasticity‐consistent robust standard errors enhances the reliability of statistical inference under potential violations of homoscedasticity assumptions.

Several limitations warrant consideration. The cross‐sectional design precludes causal inference and limits conclusions regarding directionality and temporal ordering. For instance, although educational stress was associated with higher distress, it is equally plausible that adolescents experiencing greater psychological symptoms perceive academic demands as more burdensome. The use of a convenience sample drawn from two urban colleges may also constrain generalizability, particularly to rural populations or nationally representative cohorts. Reliance on self‐reported measures introduces the possibility of reporting bias and shared method variance, and objective indicators of physical activity or academic performance were not available. Although multiple ecological domains were incorporated, other relevant determinants—including sleep quality, social media exposure, socioeconomic gradients, and prior mental health history—were not assessed and may account for additional unexplained variance. Finally, although relative importance analysis clarifies comparative variance contribution, it does not establish causal primacy, and the overall explained variance indicates that meaningful determinants remain unmeasured.

Future research should prioritize longitudinal and prospective designs to clarify developmental pathways linking educational stress, peer victimization, physical activity, and psychological distress. Nationally representative studies spanning urban and rural regions would enhance generalizability and policy relevance. Further investigation of mediating and moderating mechanisms—such as whether family cohesion buffers the effects of victimization, or whether physical activity is associated with distress through sleep, self‐esteem, or social connectedness—would deepen theoretical understanding. Intervention‐based research evaluating school‐level academic stress reduction strategies and physical activity promotion programs is also needed to strengthen translational impact. Incorporating objective behavioral assessments and multi‐informant reports may further improve measurement precision and reduce shared method bias.

## 5 Conclusions

This study demonstrates that psychological distress among Bangladeshi adolescents is associated with the convergence of educational, interpersonal, and behavioral influences embedded within their daily ecological contexts. Educational stress emerged as the most prominent correlate, underscoring the centrality of the school environment in adolescent mental health. Peer victimization and gender‐related vulnerability were also associated with elevated distress, whereas physical activity showed a consistent inverse association across depression, anxiety, and stress domains. By integrating multiple microsystem influences within a unified analytic framework and applying variance decomposition techniques, the study moves beyond isolated risk‐factor identification to clarify the relative salience of contextually grounded determinants. The findings highlight the importance of coordinated, school‐centered strategies that address academic pressure, strengthen peer safety, and promote health‐enhancing behaviors. Taken together, this ecological perspective provides direction for multi‐level preventive efforts aimed at improving adolescent mental well‐being in Bangladesh and similar LMIC settings.

## Author Contributions

**Firo Al Mamun**: conceptualization, investigation, methodology, data curation, formal analyses, writing – original draft, writing – review and editing. **Tasmin Sayeed Nodi**: investigation, methodology, project administration, data curation. **Maysa Mohamed Rabea Abdelall**: conceptualization, methodology, writing – review and editing, supervision, resources, validation. **Jyotie Malakar**: investigation, methodology, project administration, data curation, resources, conceptualization. **Susmita Hossain**: investigation, methodology, project administration, data curation, resources, conceptualization. **Md. Mehedee Hasan**: investigation, methodology, project administration, resources, data curation. **Abu Hasnat Abdullah**: investigation, methodology, project administration, data curation. **Mohammed A Mamun**: conceptualization, investigation, methodology, validation, writing – original draft, writing – review and editing.

## Funding

Dr Maysa Mohamed Rabea Abdelall is currently receiving funding support from the Princess Nourah Bint Abdulrahman University Researchers Supporting Project Number (PNURSP2026R802), Princess Nourah Bint Abdulrahman University, Riyadh, Saudi Arabia.

## Ethics Statement

The study protocol received ethical approval from the Institutional Review Board of CHINTA Research Bangladesh [Ref: CHINTA/IRB/03‐2025/3(4)] and was conducted in accordance with the ethical principles outlined in the Declaration of Helsinki. Prior to initiating data collection, official permission was obtained from the principals of the participating colleges. All participants were 17 or 18 years old at the time of the study; therefore, parental or legal guardian consent was not required under the approved ethical guidelines. Written informed consent was obtained from each participant before participation. Participation in the study was voluntary. Students were informed of their right to refuse participation or withdraw from the study at any point without academic or personal repercussions. To ensure confidentiality and anonymity, no identifying information was collected, and all responses were handled in a secure and confidential manner.

## Conflicts of Interest

The authors declare no conflicts of interest.

## Data Availability

The data that support the findings of this study are available from the corresponding author upon reasonable request.
